# Biomechanical Effects of Sandal Strap Design on Gait Kinematics and Electromyographic Activation Patterns: A Speed-Dependent Analysis

**DOI:** 10.1155/abb/8802614

**Published:** 2025-08-07

**Authors:** Bojie Xuan, Dong Sun, Dongxu Wang, Diwei Chen, Fengping Li, Yang Song, Xuanzhen Cen, Gusztáv Fekete, Monèm Jemni, Yaodong Gu

**Affiliations:** ^1^Faculty of Sports Science, Ningbo University, Ningbo, China; ^2^Ningbo No. 2 Hospital, Zhejiang Engineering Research Center for New Technologies and Applications of Helium-Free Magnetic Resonance Imaging, Ningbo, China; ^3^Department of Biomedical Engineering, The Hong Kong Polytechnic University, Hong Kong, China; ^4^Department of Materials Science and Machine Design, Széchenyi István University, Győr, Hungary; ^5^Centre for Mental Health Research in Association, University of Cambridge, Cambridge, UK

**Keywords:** biomechanics, gait, iEMG, sandals, walking condition, walking speed

## Abstract

**Background:** Sandals are widely favored for their comfort; however, their open design may reduce foot support and compromise gait stability.

**Objective:** This study examined the effects of various sandal strap configurations and walking speeds on spatiotemporal gait parameters and the integrated electromyographic (iEMG) activity of lower limb muscles.

**Methods:** Twenty-four healthy adult males (age: 25.00 ± 1.22 years; mass: 71.50 ± 11.84 kg; height: 173.50 ± 3.50 cm) participated in this study. A two-way repeated-measures ANOVA was performed to assess the effects of three footwear conditions (barefoot, Crocs strapped, and Crocs strapless) across three walking speeds (1.2, 1.6, and 2.0 m/s). Gait outcomes included step length, step width, step frequency, peak plantar loading duration, and iEMG activity of key lower limb muscles: gluteus maximus (GM), rectus femoris (RF), biceps femoris (BF), tibialis anterior (TA), and lateral gastrocnemius (LG).

**Results:** Footwear condition significantly affected step width (*p* < 0.05) and step frequency (*p* < 0.001). A significant interaction between footwear and walking speed was observed for peak plantar loading duration in both the forefoot and heel regions (*p* < 0.05). Additionally, significant differences in RF and GM iEMG activity were found between barefoot and strapped conditions (*p* < 0.05).

**Conclusions:** Strapped sandals improve plantar load distribution and gait stability by regulating step frequency and reducing lower limb muscle activation, with these effects being more pronounced at higher walking speeds, particularly during forefoot and heel loading phases.

## 1. Introduction

In recent years, sandals have gained popularity in daily life due to their open design, lightweight construction, enhanced breathability, and convenience of wear [1, 2]. Despite these benefits, sandals generally provide limited structural support, potentially influencing plantar pressure distribution, gait stability, and muscle activation patterns in the lower limbs [3–5]. Previous research has demonstrated that footwear modifications, including changes in structure and material properties, significantly impact spatiotemporal gait parameters and neuromuscular control strategies [6]. For instance, James et al. [7] reported that FitFlop sandals substantially altered gait patterns, highlighting the role of footwear structure in modulating lower limb motor control through foot stability mechanisms. However, prior studies have compared mainly distinct footwear types, with few systematic examinations of how specific structural components, particularly strap designs, affect biomechanical outcomes [8, 9].

Among various sandal types, Crocs shoes have drawn significant attention due to their distinctive foam cushioning material and adjustable heel strap system, allowing users to conveniently switch between “strapped” and “strapless” modes [10]. Specifically, the “strapped” configuration secures the heel and enhances stability, whereas the “strapless” setting emphasizes flexibility. Preliminary biomechanical research by Cham and Redfern [11] indicated that Crocs effectively reduced heel impact during slip events. In contrast, Burgess reported no significant differences in lower limb muscle activations, such as those of the rectus femoris (RF) and gastrocnemius, across various sandal-wearing conditions, highlighting the necessity of integrating spatiotemporal gait parameters for a more comprehensive biomechanical assessment [12–14]. Although studies have explored sandal performance under extreme conditions, systematic investigations into the biomechanical mechanisms by which strap designs influence gait and neuromuscular responses during routine walking remain limited [15, 16].

Additionally, walking speed is critical in modulating gait coordination and neural control, directly impacting physiological loading patterns and energy expenditure [17]. It is strongly correlated with gait stability, coordination, and muscular synergy mechanisms [18]. Yu and Kramer [17] highlighted significant changes in gait coordination and variability in response to varying walking speeds during barefoot conditions, underscoring walking speed's vital role in modulating the impact of footwear structure on gait. Consequently, evaluating sandal strap design independently of walking speed might inadequately represent the true biomechanical influences on gait performance and muscle control [19].

Addressing these research gaps, the present study examines Crocs sandals under two typical conditions (strapped and strapless) and uses barefoot walking as a control condition, employing a two-factor experimental design with three footwear conditions (barefoot, strapped, and strapless) and three walking speeds (1.2, 1.6, and 2.0 m/s) [20]. This study systematically investigates both the primary and interaction effects of sandal strap configurations and walking speeds on gait parameters and lower limb muscle activations. The objective is to elucidate the dynamic coupling between footwear configurations and walking speeds and their combined influences on gait regulation mechanisms.

By integrating structural footwear modifications with dynamic gait scenarios, this research aims to uncover the biomechanical mechanisms through which sandal strap configurations affect gait control. The findings provide scientific support for the design of functional footwear, particularly sandals intended for daily walking or rehabilitation training. By enhancing gait stability and reducing muscular load, this research offers practical implications for the prevention of gait-related injuries and the optimization of rehabilitation strategies.

## 2. Materials and Methods

### 2.1. Participants

The sample size for this study was calculated using G*⁣*^*∗*^Power 3.1 (Franz Faul, Germany), resulting in a required sample of 24 participants (effect size = 0.5, *α* error probability = 0.05) [21]. Accordingly, 24 healthy male participants (age: 25.00 ± 1.22 years; mass: 71.50 ± 11.84 kg; height: 173.50 ± 3.50 cm), all with right-leg dominance, were recruited. Inclusion criteria required no history of significant lower limb injuries within the past 6 months, no engagement in strenuous physical activity within the previous 48 h, and no musculoskeletal or neurological conditions that could affect gait performance. All participants were fully informed about the study's objectives, procedures, and potential risks and provided written informed consent before participation. Ethical approval was obtained from the Ethics Committee of Ningbo University (Approval No: RAGH20241107).

### 2.2. Experimental Protocol

**Figure 1:**
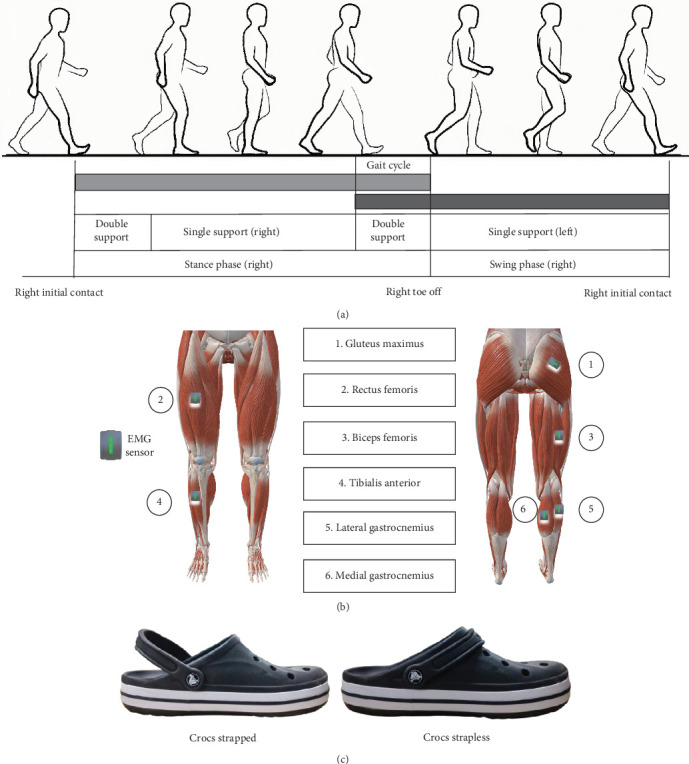
(A) Schematic diagram of the gait cycle; (B) EMG marker placement location; (C) two wearing conditions of Crocs.

Experiments were conducted in the biomechanics laboratory at Ningbo University under controlled environmental conditions. A two-factor repeated-measures design was employed, with three footwear conditions (Barefoot, Crocs strapped, and Crocs strapless) and three walking speeds (1.2, 1.6, and 2.0 m/s) (Figure 1B) [22]. The independent variables were footwear conditions and walking speeds, while the dependent variables included gait parameters and electromyographic (EMG) indicators. Before testing, participants were thoroughly briefed on the experimental protocol and given time to familiarize themselves with the tasks. Each session began with a 5-min warm-up, followed by trials randomized through computer-generated sequences to avoid order effects. A 2-min adaptation period preceded each footwear condition, and experimental data were continuously recorded for 1 min. Each condition–speed combination was repeated five times, with three representative trials used for subsequent data analysis.

### 2.3. Data Acquisition

Participants performed experimental trials in the assigned footwear conditions or barefoot, with 30-s rest intervals between trials to prevent fatigue. Gait parameters were captured using the Zebris FDM-T treadmill system (Zebris Medical GmbH, Isny, Germany).

Surface EMG signals were collected by SENIAM guidelines and anatomical landmarks, with electrodes positioned to target the gluteus maximus (GM), RF, biceps femoris (BF), tibialis anterior (TA), medial gastrocnemius (MG), and lateral gastrocnemius (LG) muscles (Figure 1B). Data acquisition was performed using a Delsys Trigno wireless surface EMG system (Delsys, Boston, USA), with electrodes placed parallel to the orientation of the muscle fibers. To minimize skin impedance, the target area was shaved and thoroughly cleaned with 75% alcohol swabs before electrode placement. EMG signals were sampled at 2000 Hz and band-pass filtered between 20 and 450 Hz. All recordings were acquired and stored in real time using EMGworks software (Delsys, Boston, USA) for subsequent analysis.

### 2.4. Data Processing

Gait parameters were extracted using the Zebris FDM-T treadmill gait analysis system, which automatically provides key spatiotemporal indicators such as step length, step width, cadence, lateral symmetry, and maximum loading durations for forefoot, midfoot, and heel regions. To ensure data stability and representativeness, initial and final gait phases were excluded, and only the stable 1-min continuous gait cycles were analyzed. Parameters were calculated as means and standard deviations for statistical analysis.

Raw EMG signals were first band-pass filtered between 20 and 450 Hz to remove motion artifacts and external noise, thereby, ensuring signal quality. The filtered signals were then full-wave rectified, converting all negative values to positive for subsequent analysis. The rectified signals were further smoothed using a fourth-order Butterworth low-pass filter with a cutoff frequency of 20 Hz to generate an envelope reflecting overall muscle activation. For data analysis, integrated electromyographic (iEMG) for each gait cycle was computed by numerically integrating the rectified envelope within the cycle, representing the area under the curve, and thus, providing a comprehensive measure of neuromuscular activation [23, 24]. To account for inter-individual variability and amplitude fluctuations across gait conditions, all EMG signals were normalized using a maximal normalization approach, whereby each data point was divided by the maximum EMG value of the respective muscle across all gait cycles for the same subject under the same condition.

### 2.5. Statistical Analysis

Data normality was assessed using the Shapiro–Wilk test, and homogeneity of variance was verified through Levene's test. A two-way repeated-measures ANOVA (footwear conditions: barefoot, strapped, strapless × walking speeds: 1.2, 1.6, 2.0 m/s) was utilized to evaluate main and interaction effects. Effect sizes were expressed as partial eta squared (*ηp*^2^) and interpreted as small (>0.02), medium (>0.13), or large (>0.26) [25]. The significance level was set at *p* < 0.05. When significant main effects were observed, pairwise post-hoc comparisons were conducted. In cases of significant interaction effects, Bonferroni-corrected post-hoc tests were applied to control for Type I errors resulting from multiple comparisons.

## 3. Results

### 3.1. Gait Performance

Table 1 reveals a significant effect of walking speed on step length (*p* < 0.001), with step length consistently increasing across all footwear conditions as speed increased. Under the barefoot condition, step length increased by 27.43 cm when speed rose from 1.2 to 1.6 m/s, followed by a further increase of 13.74 cm as speed reached 2.0 m/s. Although similar increasing trends were observed under both the strapless and strapped conditions, the differences between footwear conditions did not reach statistical significance (*p*=0.102).

In contrast, step width did not vary significantly across walking speeds (*p*=0.412). However, a significant main effect of footwear condition was observed (*p*=0.034). Specifically, compared to barefoot walking, the strapped condition consistently resulted in greater step width at all speeds, with increases of 0.76, 1.23, and 0.95 cm at 1.2, 1.6, and 2.0 m/s, respectively. Additionally, under the strapless condition, step width was also greater than barefoot walking at higher speeds (1.6 and 2.0 m/s), with respective increases of 0.50 and 0.45 cm. Although the main effect reached statistical significance, post-hoc pairwise comparisons failed to identify significant differences between specific conditions.

Cadence increased significantly with walking speed (*p* < 0.001), and a significant main effect of footwear condition was also observed (*p* < 0.001). Across all tested speeds, barefoot walking consistently resulted in a higher cadence compared to both strapless and strapped conditions. Specifically, compared to the strapless condition, barefoot walking yielded cadence increases of 4.76, 3.37, and 4.53 steps per minute at walking speeds of 1.2, 1.6, and 2.0 m/s, respectively; compared to the strapped condition, the increases were 4.36, 3.18, and 5.35 steps per minute. Post-hoc pairwise comparisons further supported these findings. At 1.2 m/s, the cadence under the barefoot condition (120.24 steps/min) was significantly higher than both the strapless (115.48 steps/min, *p*=0.024) and strapped conditions (115.08 steps/min, *p*=0.014). Similarly, at 2.0 m/s, barefoot cadence (141.27 steps/min) remained significantly greater than that of the strapless (136.74 steps/min, *p*=0.024) and strapped conditions (135.92 steps/min, *p*=0.014).

### 3.2. Maximum Loading Duration in Forefoot, Midfoot, and Heel Regions

Table 2 demonstrates a significant effect of walking speed on forefoot maximum loading duration (*p* < 0.001), with a clear trend of increased duration as walking speed increased. Under barefoot conditions, forefoot loading duration increased by 5.99% and 2.77% at moderate and high speeds, respectively, compared to the lowest speed. Footwear condition also had a significant main effect on forefoot loading duration (*p*=0.001). At 2.0 m/s, the barefoot condition resulted in significantly longer loading durations than both the strapless and strapped conditions, with increases of 2.83% and 1.91%, respectively. Furthermore, a significant interaction between walking speed and footwear condition was identified (*p*=0.030), indicating that the impact of strap configuration on forefoot loading duration varied across different speeds. Bonferroni post-hoc tests further revealed significant main effects of both walking speed and footwear condition on forefoot loading duration (*p* < 0.001). Pairwise comparisons supported the interaction effect: at 1.6 m/s, the barefoot group (81.73%) exhibited significantly longer loading durations than both the strapless group (77.67%, *p* < 0.001) and the strapped group (79.36%, *p*=0.031). Similarly, at 2.0 m/s, the barefoot group (84.50%) showed a significantly longer duration compared to the strapless group (81.67%, *p*=0.016).

In the midfoot region, increasing walking speed significantly reduced the maximum loading duration (*p* < 0.001), a trend that was consistent across all footwear conditions. Under barefoot conditions, the loading duration decreased by 7.92% and 0.13% at moderate and high speeds, respectively, compared to the lowest speed. Footwear condition also exerted a significant main effect (*p* < 0.001); at 2.0 m/s, the strapless condition resulted in significantly longer midfoot loading durations than both the barefoot and strapped conditions, with increases of 4.66% and 7.59%, respectively. Post-hoc pairwise comparisons further elucidated these group differences. At 1.2 m/s, the barefoot group (36.95%) showed significantly shorter loading durations than the strapless (46.47%, *p* < 0.001) and strapped groups (43.74%, *p*=0.048). At 1.6 m/s, the barefoot group (29.03%) remained significantly lower than the strapless group (34.62%, *p*=0.040). Similarly, at 2.0 m/s, the loading duration in the barefoot condition (28.90%) was significantly shorter than in the strapless condition (33.56%, *p*=0.004).

In the heel region, walking speed had a significant effect on maximum loading duration (*p* < 0.001), with a clear trend of reduction under both barefoot and strapless conditions as speed increased. Specifically, under the barefoot condition, heel loading duration decreased by 2.12% and 1.23% at moderate and high speeds, respectively, while under the strapless condition, the corresponding decreases were 2.91% and 0.29%. Additionally, a significant interaction was observed between walking speed and footwear condition (*p*=0.045), suggesting that the influence of strap configuration on heel loading duration varied across different walking speeds. Bonferroni post-hoc tests revealed significant main effects of both walking speed and footwear condition on heel loading duration (*p* < 0.001). Further pairwise comparisons clarified these interaction effects: at 1.2 m/s, the barefoot group (19.94%) exhibited significantly longer heel loading durations than both the strapless group (15.69%, *p*=0.016) and the strapped group (11.32%, *p*=0.001). A similar pattern was observed at 1.6 m/s, where the barefoot group (17.82%) showed significantly longer durations compared to the strapless (12.78%, *p*=0.004) and strapped groups (11.17%, *p*=0.001).

### 3.3. Muscle Activation

**Figure 2:**
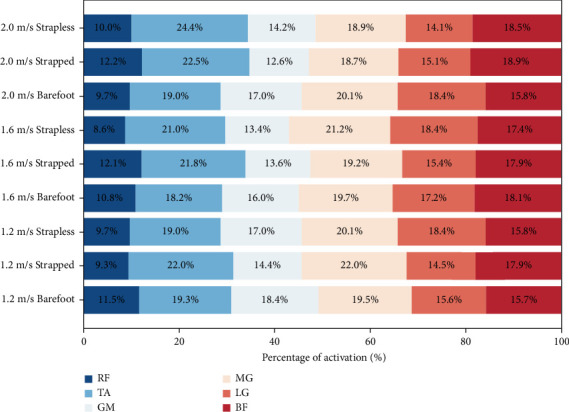
Proportion of iEMG for each muscle relative to total activation at three walking speeds.

As shown in Figure 2, at a walking speed of 1.2 m/s, significant differences in iEMG were observed in the RF between barefoot (14.60%) and strapped modes (10.30%, *p* < 0.001), as well as between strapped and strapless modes (14.05%, *p*=0.017). For the TA, significant differences occurred between barefoot (17.84%) and strapped modes (17.20%, *p*=0.002). Additionally, significant differences in GM activation were found between barefoot (19.36%) and both strapped (14.58%, *p* < 0.001) and strapless modes (14.71%, *p*=0.002).

At 1.6 m/s, the RF showed significant differences between barefoot (11.47%) and both strapped (9.04%, *p*=0.012) and strapless modes (7.90%, *p*=0.012). Similarly, significant differences in GM were noted between barefoot (15.69%) and strapped modes (14.77%, *p*=0.029).

At 2.0 m/s, only the LG exhibited a significant difference in iEMG between barefoot (15.90%) and strapped modes (13.96%, *p*=0.020).

### 3.4. Correlation Analysis Results Among Independent Variables

**Figure 3:**
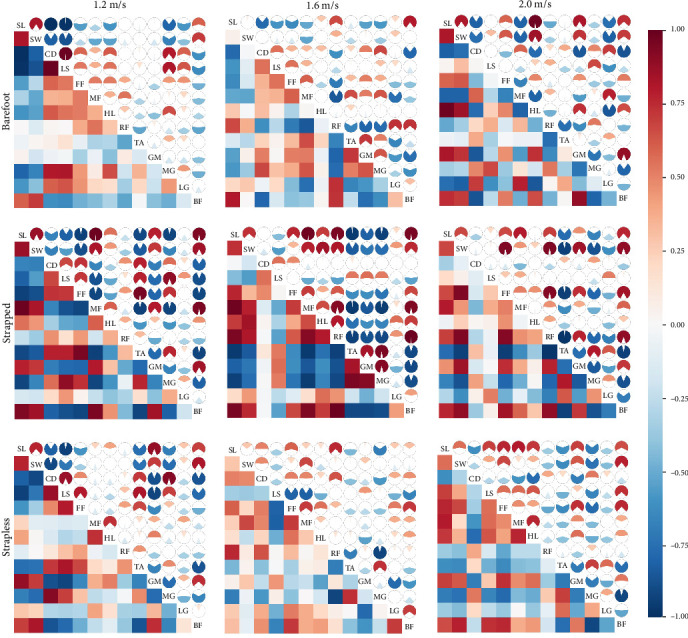
The distribution of correlation coefficients between gait parameters and muscle activation characteristics under different footwear conditions and walking speeds is shown. In the lower left of the figure, each colored square in the matrix represents the Pearson correlation coefficient between variables, with colors ranging from dark red (positive correlation), through white (no correlation), to dark blue (negative correlation). The upper right of the matrix uses pie charts to present the strength and direction of these correlations intuitively. CD, cadence; FF, time to maximum force of the forefoot; HL, time to maximum force of the heel; LS, lateral symmetry; MF, time to maximum force of the midfoot; RF, TA, GM, MG, LG, BF, IEMG contribution rates of individual muscles; SL, stride length; SW, step width.

As shown in Figure 3, under the barefoot condition, stride length was significantly associated with various gait parameters, plantar force timing characteristics, and lower limb muscle activations. At a walking speed of 1.2 m/s, stride length was positively correlated with step width (*r* = 0.82), and showed strong negative correlations with cadence (*r* = −1.00) and lateral symmetry (*r* = −0.98). Moderate negative correlations were also observed with heel time to peak force (*r* = −0.60) and MG activation (*r* = −0.80). At 1.6 m/s, stride length remained negatively correlated with cadence (*r* = −0.78), lateral symmetry (*r* = −0.60), and forefoot time to peak force (*r* = −0.64), while showing positive correlations with RF (*r* = 0.66) and BF activation (*r* = 0.80). At 2.0 m/s, stride length was positively associated with step width (*r* = 0.81), heel time to peak force (*r* = 0.97), and GM activation (*r* = 0.82), but negatively associated with cadence (*r* = −0.82) and midfoot force timing (*r* = −0.81).

Under strapless conditions, at 1.2 m/s, stride length showed strong positive correlations with step width (*r* = 0.86) and midfoot time to peak force (*r* = 0.98), but negative correlations with cadence (*r* = −0.67), lateral symmetry (*r* = −0.82), and forefoot force timing (*r* = −0.93). At 1.6 m/s, stride length was positively correlated with step width (*r* = 0.73), and with time to peak force in the forefoot (*r* = 0.76), midfoot (*r* = 0.97), and heel (*r* = 0.70), along with RF activation (*r* = 0.94). At 2.0 m/s, stride length remained positively associated with step width (*r* = 0.67), forefoot (*r* = 0.66) and midfoot (*r* = 0.79) force timing, and RF activation (*r* = 0.69), while showing a negative correlation with TA activation (*r* = −0.77).

Under strapped conditions, at 1.2 m/s, stride length was positively associated with step width (*r* = 0.77) and negatively correlated with cadence (*r* = −0.82), lateral symmetry (*r* = −0.93), forefoot time to peak force (*r* = −0.60), and TA activation (*r* = −0.76). At 1.6 m/s, stride length remained positively correlated with forefoot force timing (*r* = 0.60), as well as with RF (*r* = 0.75) and BF activation (*r* = 0.80), while the negative association with TA activation (*r* = −0.76) persisted. At 2.0 m/s, stride length was negatively correlated with cadence (*r* = −0.62) and positively correlated with lateral symmetry (*r* = 0.69), and time to peak force in the forefoot (*r* = 0.76), midfoot (*r* = 0.79), and heel (*r* = 0.71).

## 4. Discussion

This study systematically examined the interactive effects of sandal strap configurations and walking speeds on gait control mechanisms. The findings revealed that different strap configurations significantly modulate spatiotemporal gait parameters and alter the activation patterns of major lower limb muscles [18, 26]. The functional advantages of sandal straps became most evident at higher walking speeds, displaying progressively enhanced adaptive characteristics with increasing locomotor demand [17]. These findings provide novel biomechanical evidence supporting the concept of structural–functional coupling between footwear design and neuromuscular control.

Increasing walking speed resulted in significant alterations in both step length and cadence, underscoring the role of speed as an external modulator of gait rhythm [26]. This further reflects the adaptive modulation of gait rhythm mediated by sensory feedback and central nervous system integration [27, 28]. Moreover, footwear conditions had a significant impact on gait performance. Lieberman et al. [29] highlighted the different effects of barefoot and shod conditions on gait stability, particularly when footwear provides inadequate support, which results in a marked increase in step width. He proposed that individuals adopt compensatory gait strategies to enhance postural stability. Comparative analyses between barefoot and strapless conditions revealed that the absence of heel restraint leads to a significant increase in step width [30, 31]. This phenomenon likely reflects a compensatory strategy aimed at maintaining lateral stability, aligning with established biomechanical principles of gait stability, wherein an increased step width serves to expand the base of support and facilitate the redistribution of lateral forces [32, 33]. At an equivalent walking speed, barefoot gait exhibited a higher cadence than that observed under sandal conditions, possibly attributable to enhanced plantar sensory feedback, which may improve the central nervous system's capacity for more precise gait rhythm regulation [34]. The cushioning properties of soft foam soles may attenuate or delay the transmission of mechanical feedback from ground reaction forces. As this feedback is crucial for precise regulation of gait rhythm, the softness of the sole material may reduce the accuracy of gait rhythm control [35].

Liau's study investigated the effects of different walking speeds on plantar pressure distribution and reported that, compared to moderate (3.6 mph) and fast (5.4 mph) walking, slow walking at 1.8 mph may compromise postural control [36]. Analysis of plantar loading duration revealed significant main effects of both walking speed and footwear condition across various foot regions, with particularly pronounced interaction effects observed in the forefoot and heel areas [37]. As walking speed increased, the duration of forefoot loading progressively lengthened, while midfoot and heel loading durations decreased. These speed-related adaptations were most pronounced under barefoot conditions, emphasizing the distinct regional responses of the plantar surface to both locomotor demands and the presence or absence of foot support [38]. These findings suggest that, in the absence of adequate sole support, as exemplified by barefoot walking, individuals may increasingly rely on forefoot-driven propulsion to preserve gait efficiency and dynamic stability, a pattern consistent with the observed prolongation of forefoot loading duration [39].

It is important to acknowledge that, although this study primarily focused on strap configurations, the cushioning and energy-absorbing characteristics of the soft foam sole may have confounded the isolation of strap-specific structural effects [40, 41]. Future research should control for sole material properties to isolate and clarify the causal relationship between strap design and gait performance [42]. Furthermore, sandal straps enhance foot–footwear coupling, effectively minimizing foot slippage and hysteresis effects, thereby, facilitating more efficient transmission of vertical ground reaction forces [4]. This structural enhancement not only alters the spatiotemporal distribution of plantar loading but may also reduce superfluous energy expenditure, thereby, enhancing gait efficiency and offering valuable implications for functional footwear design [2, 43].

EMG analyses further elucidated the neuromuscular control strategies modulated by both footwear structure and walking speed [44]. At slower walking speeds, barefoot gait was associated with significantly elevated activation levels of the RF, TA, and GM muscles, suggesting increased muscular recruitment to maintain postural stability in the absence of structural support [12, 45]. Enhanced proprioceptive feedback from the plantar surface likely facilitated increased input from muscle spindles and cutaneous mechanoreceptors, thereby, augmenting the central nervous system's sensitivity to dynamic loading and postural modulation [46]. At higher walking speeds, sandals with straps were associated with significantly elevated RF activation, suggesting enhanced dynamic stability of the knee joint. Moreover, modulation of GM activation underscored the pivotal role of hip musculature in both forward propulsion and postural stabilization [47, 48]. Variations in LG muscle activation reflected adaptive adjustments in lower limb neuromuscular synergy, aimed at preserving overall gait coordination [49]. Additionally, Lung's research demonstrated that, compared to slow walking, both fast walking and jogging induce greater fatigue in the TA muscle, which may subsequently impair neuromuscular control mechanisms under higher-intensity gait conditions [50].

Building upon these findings, the present study further explored the interactions between stride length and key biomechanical parameters across different footwear conditions [51]. The results indicated that stride length was modulated not only by walking speed but also by distinct neuromechanical strategies associated with each footwear condition [52]. In the barefoot condition, stride length was positively associated with step width and negatively associated with cadence and lateral symmetry, indicating a potential trade-off between spatial gait expansion and inter-limb coordination in the absence of structural foot support [53]. The negative correlations observed between stride length and both heel and midfoot force timing, as well as MG activation, suggest modifications in propulsion strategies marked by a reduced reliance on posterior musculature. Under strapless conditions, stride length showed stronger correlations with delayed loading in the midfoot and forefoot regions, accompanied by reduced TA activation, particularly at higher walking speeds, indicating compromised stability and diminished dorsiflexor engagement [17]. In contrast, the strapped condition exhibited more consistent and integrated relationships among stride mechanics, plantar pressure distribution, and muscle activation, suggesting that the enhanced foot stabilization provided by straps facilitates more effective neuromechanical coordination [8, 54]. These findings underscore the critical role of footwear structure in modulating gait regulation strategies through the facilitation of dynamic neuromuscular coordination.

Significant interaction effects between strap configuration and walking speed were also identified in forefoot and heel loading durations [39]. This phenomenon likely reflects the regulatory function of strap structures in enhancing dynamic foot–shoe coupling and overall locomotor stability [32, 55]. Increased walking speeds accelerate the heel-to-toe rollover process, thereby, imposing greater stability demands on the midfoot and distal foot segments [31]. Under barefoot or strapless conditions, insufficient heel control facilitates earlier load transfer to the forefoot, thereby prolonging forefoot loading duration [56, 57]. In contrast, secure strap configurations enhance heel stability, prolong heel contact duration, promote more balanced load distribution, and improve rollover efficiency [8, 9]. This mechanism may play a pivotal role in modulating changes in forefoot and heel loading durations [58]. These findings suggest that sandal straps not only offer structural support but also facilitate the reorganization of muscular synergy, thereby promoting dynamic neuromuscular adaptations in gait regulation strategies. This deepens the theoretical understanding of the interplay between structural interventions, neuromuscular control, and motor performance [16, 59].

Although this study elucidated the effects of specific Crocs sandal strap structures and walking speeds on gait parameters and lower limb muscle activation, several limitations should be acknowledged. First, the participants were exclusively healthy adult males, which restricts the generalizability of the findings to other genders, age groups, and body types. Future research should include a broader sample to investigate potential differences in responses to footwear interventions across diverse populations. Second, all experiments were conducted using a single model of Crocs sandals, so the results are only applicable to the tested sandal type and cannot be directly extrapolated to sandals of other structures, materials, or brands. Additionally, gait experiments were performed on a Zebris treadmill. While this controlled setting improves the standardization and reproducibility of data collection, it introduces certain differences compared to overground walking. The treadmill's constant speed may alter natural gait patterns, and the slope and friction properties of the treadmill belt differ from those of typical walking environments. Furthermore, some participants had limited experience using a treadmill while wearing sandals, which may have affected their gait performance. Future studies are recommended to validate gait and neuromuscular adaptation mechanisms for different footwear types, walking speeds, and participant groups in real-world environments, utilizing wearable gait analysis systems and synchronized EMG technology to enhance the external validity and practical relevance of the findings. Lastly, finite element analysis has been extensively used in sports biomechanics to simulate the impact of equipment on human tissues [60–62]. Further studies should integrate this approach to reveal the biomechanical effects of sandal strap design on the internal mechanical states of foot, which will offer valuable insights for the design of functional footwear.

## 5. Conclusion

This study investigated the combined effects of footwear condition and walking speed on spatiotemporal gait parameters and lower limb muscle activation. Plantar loading durations differed across foot regions and were significantly affected by both factors, with pronounced interactions in the forefoot and heel. Higher speeds increased cadence and forefoot loading, particularly under barefoot conditions, whereas strapped sandals enhanced heel stability and reduced muscle demand. Correlation analyses showed that stride length was significantly associated with spatiotemporal parameters and muscle activation levels, exhibiting condition-specific patterns across different speeds and footwear modes. These findings highlight the dual role of straps in providing mechanical support and modulating neuromuscular control, offering valuable insights for the design of functional footwear.

## Figures and Tables

**Table 1:** Gait performance and stability metrics across walk conditions.

Variables	Protocol				Statistical effect		
	Speed	Barefoot	Strapless	Strapped	Speed	Strap	Speed × strap
	m/s	Mean (SD)	Mean (SD)	Mean (SD)	*p*		
Stride length (cm)	1.2	120.73 (1.99)	125.76 (2.23)	126.05 (1.77)	**<0.001**	**0.102**	0.996
	1.6	148.15 (1.86)	152.01 (2.08)	151.8 (1.70)			
	2.0	161.89 (2.04)	167.20 (1.93)	168.70 (1.68)			

Step width (cm)	1.2	12.47 (1.81)	13.23 (1.70)	12.24 (1.57)	0.412	**0.034**	0.931
	1.6	12.42 (1.91)	13.65 (1.87)	12.92 (1.84)			
	2.0	12.69 (2.17)	13.64 (2.14)	13.14 (2.10)			

Cadence (steps/min)	1.2	120.24 (1.75)	115.48 (1.43)	115.08 (1.29)	**<0.001**	**<0.001**	0.964
	1.6	129.62 (1.53)	126.25 (1.43)	126.44 (1.34)			
	2.0	141.27 (2.11)	136.74 (1.50)	135.92 (1.43)			

Lateral symmetry (mm)	1.2	−2.73 (3.08)	−2.96 (3.02)	−3.27 (3.61)	0.529	0.078	**0.126**
	1.6	−4.25 (3.38)	−1.57 (2.46)	−1.15 (2.81)			
	2.0	−3.17 (2.42)	−2.51 (2.43)	−1.98 (2.42)			

*Note:* Statistical significance was set to *p* < 0.05; the bold represents significant differences.

Abbreviation: SD, standard deviation.

**Table 2:** Time to maximum force of forefoot, midfoot, and heel (% of stance time).

Variables	Protocol				Statistical effect		
	Speed	Barefoot	Strapless	Strapped	Speed	Strap	Speed × strap
	m/s	Mean (SD)	Mean (SD)	Mean (SD)	*p*		
Forefoot (%)	1.2	75.74 (1.58)	76.17 (2.02)	67.02 (2.08)	**<0.001**	**0.001**	**0.0** **30**
	1.6	81.73 (1.73)	77.67 (1.75)	79.36 (1.70)			
	2.0	84.50 (1.63)	81.67 (1.85)	82.59 (1.52)			

Midfoot (%)	1.2	36.95 (10.28)	46.47 (11.27)	43.74 (8.17)	**<0.001**	**<0.** **001**	0.088
	1.6	29.03 (6.05)	34.62 (7.04)	34.99 (7.13)			
	2.0	28.90 (6.01)	33.56 (8.50)	25.97 (5.42)			

Heel (%)	1.2	19.94 (2.57)	15.69 (4.78)	11.32 (2.58)	**<0.001**	**<0.001**	**0.** **045**
	1.6	17.82 (4.35)	12.78 (2.43)	11.17 (2.62)			
	2.0	16.59 (4.60)	12.49 (1.72)	11.75 (2.39)			

*Note:* Statistical significance was set to *p* < 0.05; the bold represents significant differences.

Abbreviation: SD, standard deviation.

## Data Availability

All data relevant to the current study are included in the article; further inquiries can be directed to the corresponding author.
